# Inkjet-printed electrical interconnects for high resolution integrated circuit diagnostics

**DOI:** 10.1038/s44172-023-00073-4

**Published:** 2023-05-02

**Authors:** Kristof J. P. Jacobs

**Affiliations:** Interuniversity Microelectronics Centre (IMEC), Kapeldreef 75, B-3001 Leuven, Belgium

**Keywords:** Techniques and instrumentation, Microscopy, Electrical and electronic engineering

## Abstract

As semiconductors continue to shrink in size and become more three-dimensional in shape, the size of defects that can induce a failure also reduces, pushing the need for better fault isolation. The resolving capability of microscopes used in failure analysis (FA) is frequently limited by how close the microscope can be brought to the circuit under test. Accessibility is often restricted by the presence of probe needles or wire bonds that are needed to power up the device during the measurement. Here, I describe a robust, rapid and cost-effective method to overcome the contacting bottleneck by re-routing the probe pads with a low-profile redistribution layer, realized by conductive inkjet printing. I demonstrate that the method enables analytical FA with high spatial resolution on a backside power delivery network structure in combination with the optical beam induced resistance change (OBIRCH) technique. Electrical and structural characterization of the printing process are also reported.

## Abstract (web summary)

Kristof Jacobs reports a method for microscopic defect analysis of semiconductor devices during electrical stimulation, without interference by probe needles usually located in close proximity to the objective lens. His approach re-routes the probes that need to be electrically contacted during the measurement with a low-profile redistribution layer realized by conductive inkjet printing.

## Introduction

**Fig. 1:**
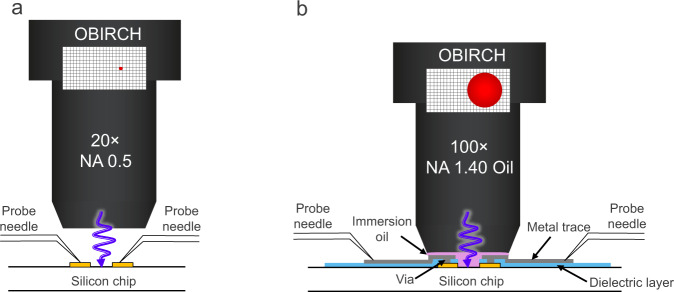
Optical beam induced resistance change (OBIRCH) principle. **a** low magnification OBIRCH imaging configuration. The large working distance objective provides opportunity to directly position the probe needles underneath it to power up the device during the test. **b** High magnification OBIRCH measurement (with small working distance objective) enabled through an on-chip redistribution layer (RDL).

Over the last 50 years, complementary metal–oxide–semiconductor technology has been following Moore’s law of device scaling where a doubling in transistor density is seen every two years^1^. The objective of scaling is to improve the performance and energy efficiency of electronic products while reducing size and cost. With geometric scaling (dimensions reduced by 30% every node), and equivalent scaling (strain engineering, high-k metal gate stacks, fin-based field-effect transistor) tapering off, the semiconductor industry has entered a new era of scaling, known as hyper-scaling. This era is driven by advances in beyond-Boltzmann transistors, embedded non-volatile memories, monolithic three-dimensional integration, and heterogeneous integration techniques^2^. The rapidly advancing integrated circuit (IC) technology brings major challenges to failure analysis (FA), a critical process where electrical and physical measurements are performed to find the cause of failures. One of the greatest challenges is the fault isolation, due to the continuing need for improved resolution and sensitivity in the analytical measurement tools to assign electrical faults to precise physical locations in the IC^3^. Techniques that enable rapid defect localization are based on optical beam (e.g. optical beam induced resistance change (OBIRCH)^4^, optical beam induced current^5^, light-induced capacitance alteration^6^, laser voltage probing^7^), electron beam (e.g. electron beam induced current^8^, resistive contrast imaging^9^, charge induced voltage alteration^10^), photon/thermal emission^11,12^, magnetic field^13^, and scanning probe^14^; important to note is that these techniques require electrical stimulation of the IC *during* the measurement. To obtain a high lateral resolution, scanning probe techniques are typically operated in the “near-field” (either in direct contact or within several nanometres), whereas optical techniques usually require an objective with a small working distance (W.D.), since resolution is directly proportional to the numerical aperture of the lens (NA ∝ 1/W.D.). Figure 1a schematically illustrates the challenge that originates from this requirement. An objective with a high magnification, and thus a small working distance, reduces the accessibility of the probe pads that needs to be electrically contacted *during* the measurement. When the probe pads are located close to the defective region, which is typical for development-oriented test structures, traditional probing or wire bonding provide limited opportunity to apply advanced resolution enhancement techniques such as numerical aperture increasing lens using solid/liquid immersion lens^15,16^, the photonic nanojet effect^17^, or near-field optical probe^18^. For optical techniques, this problem has traditionally been overcome by performing backside imaging with a solid immersion lens through a thinned and optically polished silicon substrate. Using this approach, a resolution of 122 nm was demonstrated by Serrels et al. on a 100 µm thin substrate using an illumination wavelength of 1530 nm^19^. Mechanical backside silicon thinning and polishing is not only time-consuming, but also very challenging since the sample is prone to cracking when the process is carried out by an inexperienced operator^20^. The time needed to prepare a chip for backside analysis using a selected area milling tool can easily exceed four hours (e.g., thinning to less than 100 µm, 3 mm × 3 mm cavity) due to the laborious process of thinning and polishing. The operator must continuously monitor the die shape and thickness, and adjust the milling parameters accordingly, this to compensate the warping that may occur during the thinning process. Inadequate polishing of the silicon surface will also result in surface roughness and distort the light, leading to optical losses that may compromise the backside analysis. To mitigate this distortion, it is necessary to polish the backside surface to a near-optical finish. The thickness of the remaining silicon that is needed for the backside analysis is determined by the imaging wavelength and the doping concentration of the substrate.

Moreover, since the power-delivery network of future ICs will be moved from the frontside of the chip to the backside, enabling a drastic increase in the power delivery efficiency to the IC^21^, the presence of metallization on both sides of the chip will impose limitations for traditional backside analysis approaches. As a result, the need for robust, easy-to-implement, and preferentially cost-effective probing methods is becoming more apparent to address the future FA requirements.

A solution to this problem is schematically illustrated in Fig. 1b. The approach involves the fabrication of a thin-film redistribution layer (RDL) on top of the IC to reroute the measurement pads to different locations on the chip; thereby avoiding physical interference between the electrical probes and the objective/transducer/scanning probe of the microscope. For optical techniques, the total thickness of the RDL must be less than 100 µm to enable the utilization of a liquid immersion objective for improved resolution (Olympus MPLAPON 100×O, NA: 1.4, W.D.: 0.1 mm). At these dimensions, wire bonding is not a viable probing solution anymore. This work found inspiration in the domain of advanced packaging, where RDL technology has been instrumental for the development of numerous technologies, including wafer-level packaging, fan-out packaging, through silicon via (TSV) based interposers, and chip stacks. RDLs are metal interconnects, typically from copper, that electrically connect one part of the semiconductor package to another. For multi-chiplet integrated packages, RDLs can scale from the 10 µm line-and-space range to a state-of-the-art of 1 µm line-and-space level^22^. Whereas mainstream RDL processes are carried out with wafer level infrastructure, they cannot be readily applied on singulated dies. Moreover, the need for fixed lithography masks used for mainstream RDL processes is neither a realistic option since FA requires a high degree of flexibility in trace routing. As an alternative, gas-assisted focused ion beam deposition technology could be considered as a solution^23^, however, this method is more appropriate for applications requiring sub-100-nm resolution (i.e., circuit edit of logic cells), since it would be a very slow and costly approach to fabricate RDL traces that are several millimetres long.

**Fig. 2:**
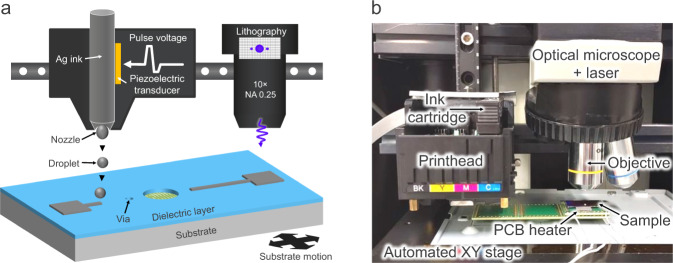
Sample preparation apparatus to enable high resolution integrated circuit (IC) diagnostics. **a** Schematic illustrating the principle of the redistribution layer (RDL) fabrication process. **b** Photograph of the sample preparation apparatus.

In this Article, a robust, easy-to-implement, and cost-effective sample preparation method is proposed and demonstrated to enable analytical FA techniques with high spatial resolution. The method offers a minimum of three times faster sample preparation time, compared to silicon thinning-based backside sample preparation, and is less susceptible to failure as the risk of sample cracking is eliminated. Moreover, the method is considered cost-effective since it can be accomplished using a combination of commonly available laboratory equipment, or by constructing a dedicated tool with a budget under 1.5 kEUR, as demonstrated in this Article, provided that an optical microscope with an automated XY table is already available. The method is based on the fabrication of an on-chip RDL and is demonstrated on a defective three-dimensional (3D) interconnect test structure. Fault isolation is carried out with the OBIRCH technique. Electrical and structural characterization results of the interconnect structures are also reported. The method is schematically illustrated in Fig. 2a, a photograph of the apparatus is shown in Fig. 2b. The method leverages inkjet printing technology as a cost-effective direct deposition technique. Inkjet printing is a well-established technique that has found application in several fields including bioengineering, 3D printing of microstructures, flexible electronics, and micromechanical and microfluidic devices^24–27^. Various inkjet printing technologies exist today, of which piezoelectric inkjet printing provides the ability to print complex inks and is considered a very mature and reliable technology. Drop-on-demand printing is a versatile technique since it is maskless and enables rapid and precise deposition of small droplets with a high degree of flexibility. As illustrated in Fig. 2a, a droplet of ink is ejected from the printhead when a voltage waveform is applied to the piezoelectric transducer. Continuous lines can be formed by arranging the ink droplets ejected from a nozzle at smaller interval than their diameter. A silver (Ag) nanoparticle ink enables the fabrication of conductive patterns. To prevent electrical shorts between the printed metallization layer and the circuitry under test, an electrical isolation layer is required, in this case, a photo-patternable polyimide layer is used. The interconnect vias between the chip and the microelectrodes are defined by laser beam lithography.

## Results and discussion

### RDL fabrication

**Fig. 3:**
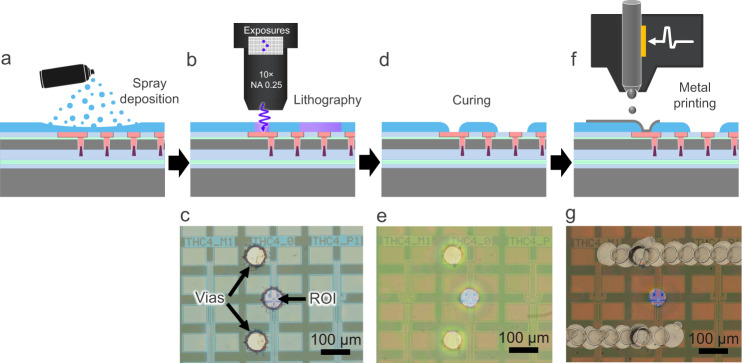
Redistribution layer (RDL) fabrication process. **a**, **b**, **d**, **f** Schematic diagrams of the four different fabrication steps applied on a backside power delivery network (BSPDN) test structure. **c**, **e**, **g** Optical micrographs of the fabrication process.

The method is demonstrated by fabricating the RDL structure on a test chip, following the scheme shown in Fig. 1b. Details about the test chip and structure are presented in the Methods section. The four-step fabrication process is schematically illustrated in Fig. 3 and is performed in a regular laboratory environment under ambient conditions. In the first step, Fig. 3a, a positive photo-patternable polyimide layer (POSITIV 20, 200 ml aerosol can, CRC Industries Europe NV, Belgium) is deposited by spray coating. Spray coating is the preferred method over spin-coating for this application, since it can be readily applied without the need for a spin coater, and arbitrarily shaped substrates can be easily coated, which is of common occurrence in FA. The polyimide layer is dried on a hot plate (MHP30, Miniware) at a temperature of 100 ˚C for 1 min. In the next step, Fig. 3b, the photo-patternable polyimide layer is structured through direct laser exposure at a wavelength of 405 nm using a defocused laser spot with a diameter of ~60 µm. The laser module used for the exposures was obtained from a Blu-ray disc reader. Three separate exposures (each of 1 min) are needed to define the region of interest (ROI) and the two vias that form the conductive pathways between the structure under test and the RDL metallization. The size of the ROI and vias can be easily varied, depending on the requirement, by controlling the laser spot defocus during the exposure. The photosensitive polyimide film is sufficiently overexposed to accommodate possible variations of the layer thickness, from 1.5 µm to 2.0 µm, that may occur during the manual spray coating process. After the exposure, the sample is developed in a sodium hydroxide solution for approximately 90 seconds. Figure 3c shows an optical micrograph of the test chip after the development.

Following the lithography step, the polyimide layer is cured to ensure the complete removal of the carrier solvent, and increase the chemical, electrical, structural, and thermal stability of the layer^28^. Moreover, the curing causes the polyimide to reflow, smoothening the via sidewalls, as illustrated in Fig. 3d, this to improve the layer conformality during metal printing. Figure 3e shows an optical micrograph of the test chip after curing, carried out on the hot plate at 240 ˚C for 10 min. No additional preparation steps regarding surface modification are taken prior to the Ag inkjet printing. In the last step, the metal electrodes are deposited by the conductive inkjet printing technique, as schematically illustrated in Fig. 3f. In this work, a Ag-based ink is chosen since it offers high electrical conductivity, low tendency toward oxidation, and generally has a high chemical stability^29^. However, other types of conductive inks such as copper inks, carbon nanotube inks, conductive polymer inks, carbon/graphene inks may also be considered as viable alternatives^30^. The printing is carried out with a printhead from a low-cost desktop inkjet printer (Expression Home XP-2155, Epson).

Good results on the printing of conductive patterns using low-cost desktop inkjet printers have previously been reported by several research groups^31–34^. In this work, the original ink cartridge is refilled with a nanosilver ink (DM-SIJ-3201, Dycotec, UK). A controller, based on the Arduino Due microcontroller board, has also been developed to drive a single nozzle of the printhead and dispense individual ink droplets following a drop-on-demand scheme. An alignment between the printhead and imaging system is carried out before each print task. To enable stable printing and avoid possible merging and bulging phenomena, which is typically observed when printing on hydrophobic surfaces, an interlacing printing scheme is applied, and the sample is heated to 70 ˚C using a small (20 mm × 20 mm) printed circuit board (PCB) meander heater^35,36^. Figure 3g shows an optical micrograph of the test chip at the end of the metal printing process. The left electrode is printed with a drop spacing of 60 µm in the direction from left-to-right during the first pass and from right-to-left during the interlacing pass; the opposite is done for the right electrode. By adopting this printing scheme, it is possible to work around the ‘first drop problem’, a well-known phenomenon in the field of inkjet printing, where the first ejected drop is often different to the subsequent drops, in terms of morphology, trajectory, and mass, impacting the dispensing accuracy. This phenomenon occurs due to the evaporation of the volatile ink components at the nozzle orifice during the non-jetting period^37^. The overlap between the printed probe pads and the interconnect lines ensures that the first-drop problem has no negative impact on the final print quality. The width of the printed interconnect line is ~70 µm. The fabrication is completed by sintering the sample on a hot plate at a temperature of 130 ˚C for 15 min, to minimize the electrical resistance of the inkjet-printed interconnects. The entire sample preparation process can be completed in under 80 min. Given the need for elevated temperatures in the current RDL fabrication process, especially during the polyimide curing stage, it is essential to consider the potential impact it may have on the defect. One solution that may address this concern is to substitute the polyimide with one that requires a lower curing temperature. Another promising approach that may offer a solution is to consider a fully inkjet-printed realization whereby an ultraviolet-curable hybrid polymer ink is used for the fabrication of the dielectric layer^38^. However, it is expected that such implementation may prove to become more challenging for tasks that require the realization of much smaller vias. This is due to the fact that a very high level of drop precision and accuracy is required from the inkjet deposition system to position the very small vias in the desired locations. Finally, it should be noted that for the present process, the adhesion of the Ag layer to the polyimide showed a good performance as it could not be separated by the Scotch-tape pull test.

### High-resolution OBIRCH

**Fig. 4:**
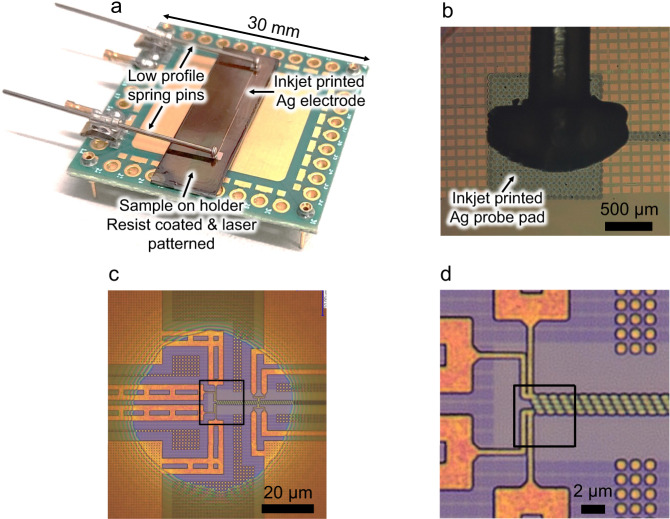
Test chip upon completion of the sample preparation process. **a** Photograph of the test chip mounted on a printed circuit board (PCB) holder, before the optical beam induced resistance change (OBIRCH) analysis. The size of the PCB is 30 mm × 30 mm. **b** Optical micrograph showing a close-up view of a spring pin in contact with a printed probe pad. **c** Optical micrograph of the region of interest (ROI) showing the presence of the aperture in the polyimide layer. **d** Optical micrograph showing a zoomed-in view of the backside power delivery network (BSPDN) chain structure, indicated by the black square in the previous subfigure.

Since 1993, OBIRCH has been an indispensable optical fault isolation technique in the semiconductor industry used both on test structures and final products^39^. The technique uses a scanning laser beam to induce a local temperature gradient in the IC and the heating-induced change in the electrical resistance is detected by a meter. The technique can detect voids in metal lines, voids underneath vias, silicon nodules, high resistivity regions, as well as current paths, analyse abnormal I_DDQ_ ICs, and more^40^. Figure 4a shows a photograph of the sample that was prepared for the high-resolution OBIRCH analysis. The sample is mounted on a small PCB carrier (30 mm × 30 mm) and electrical contacts are made to the inkjet-printed probe pads using low-profile spring pins. The force exerted by the spring pins ensures that the sample remains in position on the PCB carrier, since no adhesive is used, the sample can be mounted and replaced in a quick and easy manner. A close-up view of a spring pin contacting the inkjet-printed probe pad is shown in Fig. 4b. The printed interconnect lines are 8 mm long and the printed probe pads are 1.2 mm × 1.2 mm in size. The total printing time of the RDL structure is 20 min. It is expected that the printing time can be further reduced, since the printing is currently limited by the travel speed of the translation stage, not the printhead itself. Figure 4c shows an optical micrograph of the opened-up ROI in the polyimide layer, and a zoomed-in view of the interconnect test structure is shown in Fig. 4d.

**Fig. 5:**
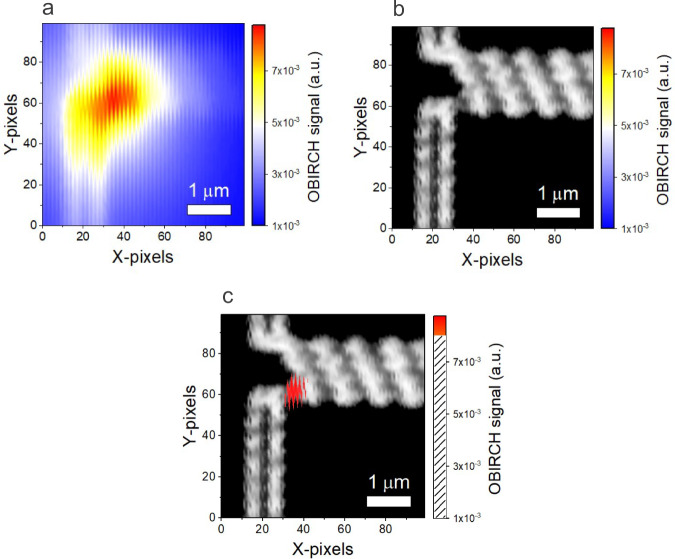
Optical beam induced resistance change (OBIRCH) measurement result on a backside power delivery network (BSPDN) test structure. **a** OBIRCH image of the region outlined by the black square in Fig. 4c. **b** Corresponding pattern image. **c** OBIRCH image superimposed on the pattern image. Thresholding is done on the OBIRCH data, by applying transparency to the values in the shaded region of the colour scale.

Figure 5a shows the OBIRCH image of a 100 × 100-pixel area, measured in the centre location of the ROI, as outlined by the black square in Fig. 4d. Figure 5b shows the corresponding pattern image. The high imaging resolution is evident in that the individual 210 nm wide metal segments and interconnect lines can be resolved in the pattern image. According to the Rayleigh criterion^41^, the optical resolution is limited to ~180 nm for this system. Both images are superimposed in Fig. 5c with thresholding applied on the OBIRCH data to highlight the centre of the signal spot. The OBIRCH and laser microscope images are captured simultaneously in less than 1 min. Details about the OBIRCH measurement setup are presented in the Methods section. A signal spot is clearly observed at the terminal of the test structure, identifying the location of a defect. Transmission electron microscopy analysis revealed the presence of a high resistance region between the nanoscale TSV (n-TSV) and the buried power rail (BPR). The results are presented in the Methods section. The observed broadness of the OBIRCH spot can be attributed to a thermal spreading effect, which is inevitable, since the OBIRCH technique is based on laser heating. This effect can possibly be further reduced by increasing the pulsing frequency of the laser^42^.

Promising approaches to further increase the optical resolution of the OBIRCH method, beyond the diffraction limit of light, include the use of a near-field optical probe (NF-OBIRCH) instead of a laser beam as the heat source, enabling a spatial resolution of 50 nm^43^, and the application of super-resolution techniques, which can provide a defect localization resolution up 18-times smaller than the diffraction limited probe spot^44,45^. Moreover, the very thin RDL material stack, less than 3 µm, is appealing for applications that require the laser spot to be focused several micrometres deep inside the chip. This is especially advantageous when analysing wafer-to-wafer (W2W) hybrid bonding interconnects that are formed between two silicon substrates. With a 100× objective having a W.D. of 0.1 mm, this remains well within reach when the top substrate is rather thin (e.g., 5 µm). The method has now been incorporated into the standard FA flow for locating resistively failed BSPDN and W2W interconnects and has shown good reproducibility. To ensure optimal outcomes, it is advisable to (1) verify the via openings in the photoresist post-development, but pre-curing, and repeat lithography if needed, and (2) conduct a nozzle check and alignment on a non-critical area of the chip before commencing the printing of the Ag lines and pads.

### Electrical and structural characterization

**Fig. 6:**
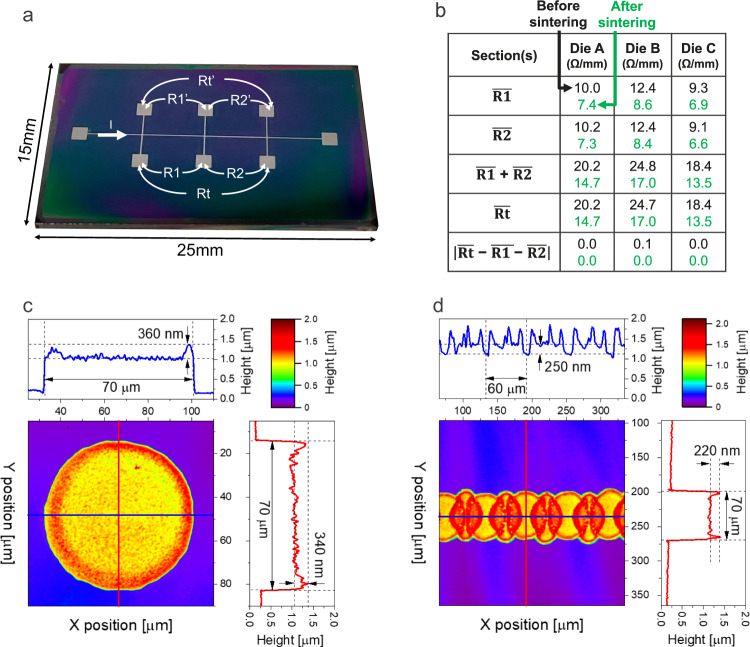
Electrical and topographic characterization of silver ink printed on a cured polyimide layer. **a** Photograph of a printed bridge structure for electrical resistance measurement. **b** Electrical measurement data of the bridge structure. **c**, **d** Interferometric images of a printed dot and line, respectively. The line profiles, horizontal in blue and vertical in red, show the height variation across the dot and line.

It is evident that the printed RDL must have a low ohmic resistance and a low leakage current to maximize the OBIRCH signal strength. The electrical properties of the inkjet-printed interconnects have been characterized with dedicated test structures. Figure 6a shows a photograph of the printed test structure to determine the resistance per unit interconnect length. The structure is designed to enable a four-probe measurement, this to exclude the effect of parasitic cabling and contact resistances from the measurement. The accuracy of the measurement is further improved by taking the average of R and R’. The fabrication flow for the test structure is identical as shown in Fig. 3, but without the lithography step, it not being required. Three samples were prepared, and the measurements were carried out with a Keithley 2450 source-measure unit instrument. The electrical measurement data, in resistance per mm length, is presented in tabular form in Fig. 6b. The measured values before and after sintering are shown in black and green, respectively. Between the three samples, the extracted resistance per unit length is found to vary from 9.1 Ω/mm to 12.4 Ω/mm before sintering, and the values lowered to 6.6 Ω/mm and 8.6 Ω/mm during sintering. For a ~ 70 µm wide interconnect line, a minimum sheet resistivity value of ~0.6 Ω/□ is calculated from the data set. From these measurements, it is found that the RDL adds a parasitic resistance of ~140 Ω (i.e., 70 Ω for each electrode) to the measurement circuit. Since this resistance is significantly less than the resistance of the measured structure, in this case 10.2 kΩ, very little impact on the OBIRCH measurement is expected, with less than 1.5% of the source voltage being dropped across the RDL. Printing several lines in parallel and using a multi-pass printing approach to widen and thicken the interconnect lines can be considered advantageous for structures that require a lower interconnect line resistance and higher operating current (e.g., power electronic devices). With a second test structure, the performance of the polyimide dielectric layer was evaluated with respect to leakage current. The test structure consists of isolated probe pads (1.2 mm × 1.2 mm) that were printed on a heavily p++ doped silicon substrate coated with the polyimide dielectric. Measuring the leakage current through the dielectric layer involves conducting a current–voltage sweep between the printed pad and the substrate. The measurement is carried out for three pads that are located next to one another. Supplementary Fig. 1 depicts the test structure and setup used for taking the measurements. An aperture with a diameter of ~100 µm was fabricated at the centre of the sample, this to enable an accurate measurement of the polyimide layer thickness using a stylus profilometer (DektakXT, Bruker). With this method, a layer thickness of 1.9 µm was measured. From the current–voltage (*I*–*V*) characteristics, a leakage current density of less than 35 pA/mm^2^ was measured at positive and negative biases of 10 V, for all three pads. Since the total area of the RDL (two probe pads and two interconnect lines) is ~4 mm^2^, the RDL can add a parasitic DC leakage current up to ~140 pA to the measurement circuit when operating at a bias below 10 V. Whereas the low leakage DC current does not pose an immediate concern to the OBIRCH measurement, it is crucial to note that many modern failure localization techniques rely on detecting time-varying signals in the several kHz to MHz range. Therefore, the introduction of a RDL directly on top of the on-chip back-end-of-line metallization may lead to signal integrity concerns such as transmission loss and crosstalk between the printed metallization structure and signal lines. To reduce crosstalk, several approaches can be considered, including minimizing the overall area of the printed Ag metallization pattern, increasing the thickness of the dielectric layer, routing the RDL to minimize overlap with critical nets, and operating at a lower detection frequency. This will be investigated in future work.

Regarding the structural characterization, the height profile of the printing process was measured using white light interferometry (Wyko NT9100, Veeco). Figure 6c shows the surface topography image of a single printed dot, with corresponding horizontal and vertical line profiles through its centre. The dot has a diameter of 70 µm. The edges of the dot show a distinct ‘ridge’, ~350 nm in height, from the so-called ‘coffee stain effect’, first described by Deegan et al.^46^. The effect occurs due to increased rate of evaporation of liquid at the drop edges, setting up an internal flow that deposits solute at the edges. The ring stain effect is commonly considered a negative effect in the field of inkjet printing, causing print non-uniformity, and several studies have developed means to prevent this effect, including decreasing the substrate temperature to reduce edge evaporation^47^, or through ink reformulation^48^. However, rings formed from metallic nanoparticle inks may also have a beneficial impact since they have been shown to be densely packed and capable of conductivities equivalent to 14% of bulk values without additional sintering^49^. A multi-layer approach can also be an alternative to mitigate the coffee ring effect and reduce the thin film resistance. Nonetheless, this approach inevitably entails additional printing time as a trade-off. To quantify the overall print thickness, the height profile of a line has been measured. It is not possible to accurately measure the thickness from a single dot with the interferometry method, due to the lack of a reference plane, given that the Ag and polyimide layer are dissimilar materials, introducing a phase shift error in the reflected light. Since the dots are overlapping in the printed line, shown in Fig. 6d, this phase error can be effectively excluded from the measurement. The measured profile data indicates a single layer thickness of 250 nm, which is confirmed by a stylus profilometry measurement conducted on a single droplet, as shown in Supplementary Fig. 2.

## Conclusions

Inkjet-printed conductive tracks, used to reroute probe pads, have shown a promising pathway to further enhance the imaging resolution of electrical fault isolation techniques that are commonly hampered by the restricted working distance when using high magnification microscope objectives. It also avoids the need for conducting backside analysis, which is a known challenging process. The method was demonstrated for the localization of a high resistance failure on a 3D interconnect test structure using the OBIRCH technique. Although the method has demonstrated favourable outcomes on test structures, its efficacy on intricate product ICs is yet to be determined. The author hopes that this research will encourage further investigation and improvements, as well as the exploration of alternative and/or complementary methods.

## Methods

**Fig. 7:**
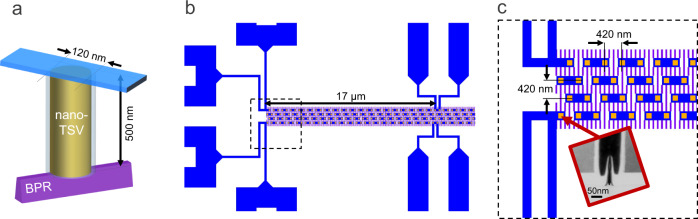
Backside power delivery network (BSPDN) test structure. **a** Schematic diagram of a nanoscale through silicon via (n-TSV) interconnecting a buried power rail (BPR) to a backside metal 1 line. **b** Layout of the BSPDN chain structure, composed of two interwoven chains. The length of the daisy chain is 17 µm. **c** Zoomed-in view of the layout region indicated by the black dotted square in the previous subfigure. The n-TSVs, BPR, and backside metal 1 segments are coloured in orange, purple, and blue, respectively. The inset shows a transmission electron microscopy cross-section image of the interface between the first n-TSV and BPR.

The sample preparation and OBIRCH methods are demonstrated on a test structure that finds use in the development of buried power rail (BPR) and nanoscale TSV (n-TSV), targeting backside power delivery network (BSPDN) application. Figure 7a shows a schematic diagram of a n-TSV interconnecting a BPR to a backside metal 1 line. The BPR power rail is 32 nm wide, the diameter of the n-TSV is 120 nm, and the thinned silicon thickness is 500 nm. Electrical measurements and simulations have shown that a tungsten filled n-TSV typically exhibits a nominal electrical resistance of 30 Ω, whereas the tungsten filled BPR has a much higher resistance of 220 Ω/µm. The main contributor to the overall chain link resistance therefore comes from the BPR segment, which spans 420 nm. Recent findings indicate that incorporating Ruthenium into the BPR can lead to a further reduction in resistance to 60 Ω/µm^50^. Defective TSVs with limited contact to the BPR have displayed resistance values ranging from a few ohms to several kiloohms.

Detailed information about the fabrication process and structure are reported elsewhere^50^. The layout of the test structure is shown in Fig. 7b. Each chain consists of multiple interconnect links that are connected in series, each link being made of one n-TSV connected to one backside metal 1 line segment at its top and one BPR segment at its bottom, following the scheme shown in Fig.7a. This structure provides opportunity to test multiple electrical connections in a simple pass/fail test. A daisy chain block containing 40 of these links was found to be defective, since the measured resistance was 4 kΩ larger than the yielding chains having a nominal resistance of 6 kΩ. The defective chain failed from limited contact between the n-TSV and BPR, leading to high resistance, as revealed in the transmission electron microscopy image of Fig. 7c.

**Fig. 8:**
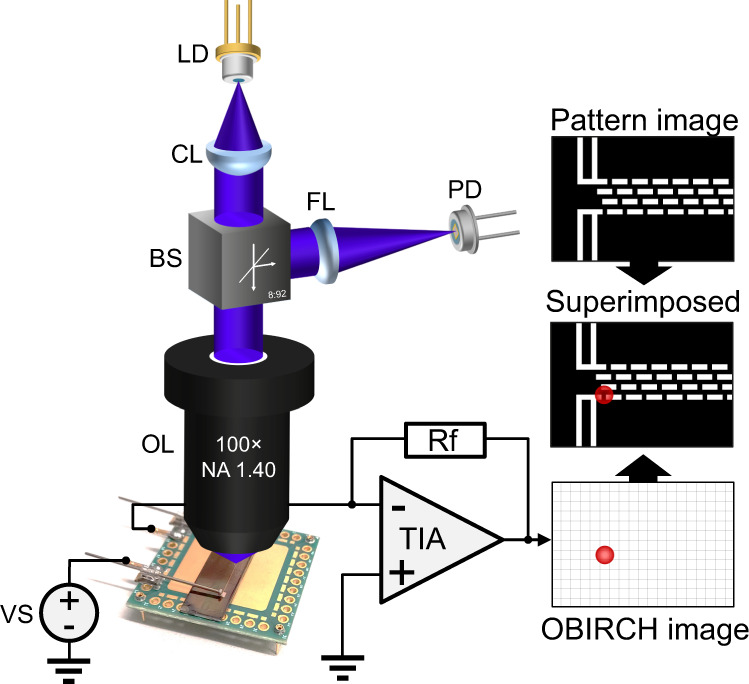
Optical beam induced resistance change (OBIRCH) measurement set-up. Schematic of the OBIRCH apparatus. LD laser diode, CL collimating lens, BS beam splitter, FL focusing lens, PD photo diode, OL objective lens, VS voltage source, TIA transimpedance amplifier, Rf feedback resistance.

A dedicated experimental setup was developed to explore the application of high-resolution OBIRCH with near-ultraviolet excitation light. Figure 8 shows a schematic diagram of the measurement system. A 405 nm laser diode from an optical module of a Blu-ray writer (BDR-212DBK, Pioneer) was used for the light source. The laser diode was driven by a laser diode controller (LDC502, Stanford Research Systems) in a pulsed current mode with a peak current of 26 mA (peak optical power of ~50 µW at the sample surface), and a modulation frequency of 50 kHz. The laser beam is focused to a focal spot with a high NA objective (Olympus MPLAPON 100×O, NA: 1.4, W.D.: 0.1 mm) and is optically coupled to the sample using immersion oil (*n* = 1.518, Olympus Type F). The pattern image is acquired by redirecting a portion of the reflected light from the sample surface, using a pellicle beam splitter with a split ratio of 10:90, to a Si-based photodetector (FDS100, Thorlabs). The photocurrent is measured with a source-measure unit instrument (Model 2450, Keithley). Imaging is performed by maintaining the position of the laser beam fixed, while raster scanning the sample that is mounted on a high-precision XY piezo stage (PIHera P-628.1, Physik Instrumente). The positioning of the stage is triggered at a frequency of 400 Hz. The step size of the XY stage is set to 50 nm and a field of view of 5 µm × 5 µm is generated for a 100 × 100-pixel scan. The electrical measurement of the OBIRCH analysis is performed at a bias of 100 mV and the current signal is amplified by a transimpedance amplifier. The signal from the transimpedance amplifier (OPA637, Texas Instruments) is demodulated by a lock-in amplifier (SRS865, Stanford Research Systems) which is synchronised to the laser diode controller. The OBIRCH image is superimposed on the pattern image in software.

### Supplementary information

Supplementary Information

## Data Availability

The data that support the plots within this paper and other findings of this study are available from the author upon reasonable request.
