# Supplementary material for: Inkjet-printed electrical interconnects for high resolution integrated circuit diagnostics
Source: Commun Eng. 2023 May 2;2:21. doi: 10.1038/s44172-023-00073-4 (PMC10955959; doi:10.1038/s44172-023-00073-4)
Supplement: Supplementary file 1 — Supplementary Information [file 44172_2023_73_MOESM1_ESM.pdf]

Inkjet-printed Electrical Interconnects for High Resolution Integrated Circuit Diagnostics

Supplementary Information

Kristof J.P. Jacobs

Imec, Kapeldreef 75, B-3001 Leuven, Belgium

Correspondence: K.J.P. Jacobs (kristof.j.p.jacobs@imec.be)

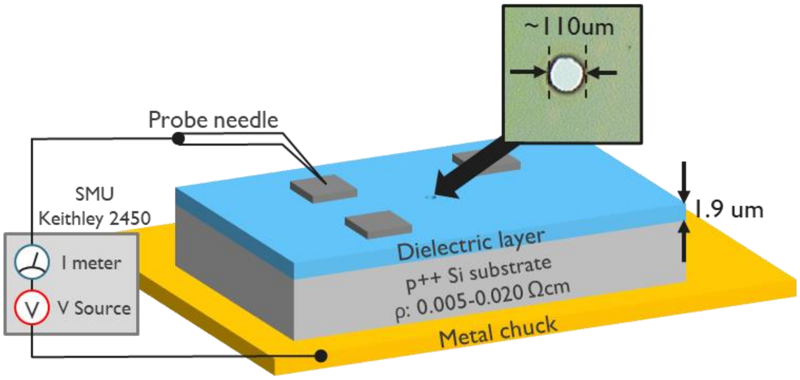

Supplementary Figure 1. **Leakage current measurement**
Schematic diagram of the test structure and experimental arrangement employed to measure the leakage current. Three probe pads, each measuring 1.2 mm × 1.2 mm, were printed on a polyimide layer using inkjet printing. The central aperture in the sample allowed for precise measurements of the polyimide layer thickness via a stylus profilometer (DektakXT, Bruker). For the current-voltage (I-V) measurements, a Keithley 2450 source measure unit (SMU) was utilized. The leakage current is measured between the printed pad and the highly doped substrate.

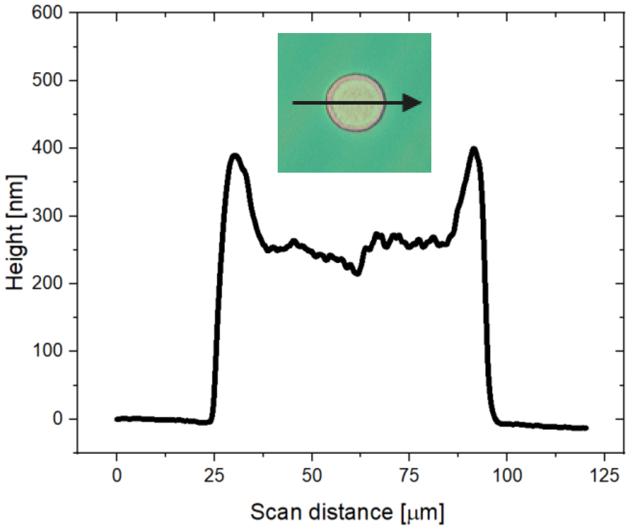

Supplementary Figure 2. **Droplet thickness measurement**
Stylus profilometry of a single Ag droplet printed on the cured polyimide layer. The inset shows an optical micrograph of the droplet and the scan direction is indicated by the arrow.
